# SC66 inhibits the proliferation and induces apoptosis of human bladder cancer cells by targeting the AKT/β‐catenin pathway

**DOI:** 10.1111/jcmm.17005

**Published:** 2021-10-22

**Authors:** Wu Chen, Sheng Zhao, Weimin Yu, Ting Rao, Yuan Ruan, Shaoming Zhu, Yuqi Xia, Hongfei Song, Fan Cheng

**Affiliations:** ^1^ Department of Urology Renmin Hospital of Wuhan University Wuhan China; ^2^ Department of Urology Qianjiang Central Hospital Qianjiang China

**Keywords:** AKT, apoptosis, bladder cancer, cell cycle, epithelial‐mesenchymal transition, SC66, β‐catenin signalling pathway

## Abstract

Bladder cancer (BC) is a major disease of the genitourinary tract, and chemotherapy is one of the main treatments commonly used at present. SC66 is a new type of allosteric AKT inhibitor that is reported to play an effective inhibitory role in the progression of many other types of tumours, but there is no reported research on its role in BC. In this study, we found that SC66 significantly inhibited the proliferation and EMT‐mediated migration and invasion of T24 and 5637 cells. In addition, experiments confirmed that SC66 achieved its antitumour effect by inducing cell apoptosis and affecting the cell cycle. Luciferase assays confirmed that SC66 exerted an antitumour effect through the AKT/β‐catenin signalling pathway, and this inhibitory effect was reversed after the addition of the β‐catenin signalling pathway activator, CHIR‐99021. In addition, animal studies have shown that, compared with the control group, the experimental group with SC66 intraperitoneal injection showed significantly reduced the tumour weight and volume in nude mice with T24 tumours and that SC66 combined with cisplatin achieved better inhibition on tumours. Western blot analysis and immunohistochemistry staining confirmed that SC66 inhibited the EMT process in vivo and induced apoptosis through the AKT/β‐catenin signalling pathway. In conclusion, our study demonstrated that SC66 exerts a significant antitumour effect through the AKT/β‐catenin signalling pathway, thereby providing a new potential treatment for BC.

## 1 INTRODUCTION

Bladder cancer (BC) is one of the 10 most common cancers in the world, and it caused more than 210,000 deaths in 2020.^1^ Due to factors, such as smoking and occupational exposure to various chemical carcinogens, the incidence of BC is gradually increasing.^2^, ^3^ At present, surgery, immunotherapy, radiotherapy and adjuvant chemotherapy are considered the main methods for the treatment of BC.

As a highly effective anti‐cancer drug, cisplatin has been proved to have great potential in the treatment of various cancers, including urological cancers, head and neck cancers and thoracic cancers. After internalization in cells, due to the aquation of one of the two chloride leaving groups, cisplatin is stimulated and can be covalently bound to DNA, which subsequently activates many molecular mechanisms involving DNA modification, cell cycle arrest and apoptosis to exert its anti‐cancer effect.^4^, ^5^, ^6^, ^7^ In addition, damage to important organelles such as the endoplasmic reticulum and mitochondria is another important mechanism of cisplatin's anti‐cancer effect. However, the abnormal activation of autophagy, unfolded protein response and other protective processes promotes the chemical resistance of cisplatin and limits its functions.^8^, ^9^

Therefore, it is extremely urgent to explore the molecular mechanisms in the progression of BC and identify new and effective treatments.

Many studies have confirmed that the phosphatidylinositol‐3 kinase (PI3K)/AKT signalling pathway plays a vital role in regulating cell proliferation, differentiation and survival.^10^, ^11^, ^12^ Abnormal activation of the PI3K/AKT signalling pathway plays an important role in the progression of many human cancers.^13^, ^14^, ^15^ AKT is a serine/threonine kinase that is activated by phosphorylation. Activated AKT regulates cell proliferation, differentiation and apoptosis through multiple downstream targets, such as mTORC1, GSK‐3β and CASP9. Therefore, AKT is also regarded as a key regulator of the PI3K/AKT signalling pathway.^16^, ^17^, ^18^

In recent years, many studies have confirmed that AKT‐targeted therapy significantly inhibits the progression of many cancers, including BC.^19^, ^20^ Therefore, drug research targeting AKT has also become an important focus in the treatment of BC.

SC66 is a new type of allosteric AKT inhibitor. Jo et al.^21^ showed that SC66 inhibits AKT activation by interfering with the binding of the pleckstrin homology domain to phosphatidylinositol‐3,4,5‐triphosphate and directly promotes AKT ubiquitination to enhance PI3K inhibition‐mediated antitumour effects. In recent years, an increasing number of studies have demonstrated that SC66 plays an inhibitory role in the development of many cancers. Yeying Liu et al. reported that SC66 mediates the apoptosis of colon cancer cells by targeting AKT.^22^ Xu et al.^23^ confirmed that SC66 inhibits the proliferation, migration and invasion of renal cell carcinoma cells by targeting AKT. Another report has shown that SC66 inhibits the proliferation of human glioblastoma and induces apoptosis in tumour cells by inhibiting AKT.^24^

However, there is still no research on the role of SC66 in BC. Thus, the present study investigated the specific role of SC66 in BC and its molecular mechanism.

## 2 MATERIALS AND METHODS

### 2.1 Drugs and antibodies

SC66 (purity of 99.88%) was purchased from Selleck (Cas: 871361‐88‐5, China) and dissolved in dimethyl sulfoxide (DMSO) to get to the required concentration. CHIR‐99021, a selective GSK‐3 inhibitor (purity of 99.76%), was purchased from MedChemExpress (Cas: 252917‐06‐9, China). The following antibodies were used in the present study: P‐AKT (66444‐1‐Ig, Proteintech), AKT (60203‐2‐Ig, Proteintech), P‐GSK‐3β (#9323, CST), GSK‐3β (#9832, CST), P‐β‐catenin (#9567, CST), β‐catenin (#8480, CST), SNAI1 (13099‐1‐AP, Proteintech), MMP2 (10373‐2‐AP, Proteintech), E‐cadherin (20874‐1‐AP, Proteintech), vimentin (10366‐1‐AP, Proteintech), cyclin D1 (60186‐1‐Ig, Proteintech), Bcl‐2 (#15071, CST), Bax (60267‐1‐Ig, Proteintech), cleaved‐Caspase‐3 (#9664, CST) and GAPDH (60004‐1‐Ig, Proteintech). More detailed antibody information is shown in Table 1.

**TABLE 1:** Details of the antibodies

Antibodies	Company	Catalogue number	Host	Dilutions
Phospho‐AKT	Proteintech	66444‐1‐Ig	Mouse	1:4000
AKT	Proteintech	60203‐2‐Ig	Mouse	1:4000
Phospho‐GSK‐3β	Cell Signalling Technology	#9323	Rabbit	1:1000
GSK‐3β	Cell Signalling Technology	#9832	Mouse	1:1000
Phospho‐β‐catenin	Cell Signalling Technology	#9567	Rabbit	1:1000
β‐catenin	Cell Signalling Technology	#8480	Rabbit	1:1000
SNAI1	Proteintech	13099‐1‐AP	Rabbit	1:1000
MMP2	Proteintech	10373‐2‐AP	Rabbit	1:1000
E‐cadherin	Proteintech	20874‐1‐AP	Rabbit	1:5000
Vimentin	Proteintech	10366‐1‐AP	Rabbit	1:4000
Cyclin D1	Proteintech	60186‐1‐Ig	Rabbit	1:5000
Bcl‐2	Cell Signalling Technology	#15071	Mouse	1:1000
Bax	Proteintech	60267‐1‐Ig	Mouse	1:5000
cleaved‐Caspase‐3	Cell Signalling Technology	#9664	Rabbit	1:1000
GAPDH	Proteintech	60004‐1‐Ig	Mouse	1:20000

### 2.2 Cell culture

Cells were cultured in a humidified atmosphere containing 5% CO_2_ at 37°C using RPMI‐1640 medium (Invitrogen) supplemented with 10% foetal bovine serum (FBS; Hangzhou Sijiqing Biological Engineering Materials Company) and 1% penicillin‐streptomycin (Life Technology).

### 2.3 Cell counting kit‐8 (CCK‐8) cell viability assay

Cell Counting Kit‐8 (Dojindo) was used to detect cell viability according to the manufacturer's instructions. T24 and 5637 cells were seeded in 96‐well plates at a density of 5000 cells/well and treated with SC66 at 0, 2, 4, 6, 8, 10, 12, 14, 16 and 18 µmol/L for 24 h. Subsequently, 10 μl of CCK‐8 was added to the cells and incubated at 37°C for 1 h. A microplate reader (Bio‐Rad Laboratories, Inc.) was used to measure the absorbance of each well at 450 nm. Three independent experiments were conducted in each group.

### 2.4 Colony formation assay

T24 and 5637 cells were seeded in a six‐well plate, cultured until 70% confluency and then treated with SC66 (0, 5 and 10 µmol/L) for 24 h. After digesting and resuspending the cells in the wells, cells were seeded in a new six‐well plate at a density of 2 × 10^3^ cells/well and cultured at 37°C for 2 weeks until single‐cell colonies were formed. After washing three times with phosphate‐buffered saline (PBS), cells were fixed with 4% paraformaldehyde for 15 min and stained with 0.5% crystal violet solution for 15 min. After washing with PBS, cells were dried in air, and the number of colonies was counted manually.

### 2.5 5‐Ethynyl‐2′‐deoxyuridine (EdU) staining

First, T24 and 5637 cells were seeded in a six‐well plate, cultured until 70% confluency and then treated with SC66 (0 and 10 µmol/L) for 24 h. EdU working solution was added to each well at a final concentration of 20 µM. After coincubation for 2 h, cells were fixed with 4% paraformaldehyde for 30 min. An EdU labelling/detection kit (RiboBio) was used to stain the cells according to the manufacturer's instructions. After washing three times with PBS, nuclei were labelled with DAPI for 30 min in the dark. Finally, EdU‐positive cells were observed by fluorescence microscopy (Olympus).

### 2.6 Wound‐healing assay

T24 and 5637 cells were seeded in a six‐well plate with RPMI‐1640 medium containing 10% FBS until cells reached 80% confluency. Cells were scraped with a sterile pipette tip to form a straight wound line. After washing three times with PBS to remove floating cells, serum‐free RPMI‐1640 medium and different concentrations of SC66 (0, 5 and 10 µmol/L) were added for further culture. A microscope (Olympus IX73) was used to acquire images at 0, 24 and 48 h. Finally, ImageJ software was used to measure and calculate the percentage of wound‐healing area at different time periods.

### 2.7 Transwell migration and invasion assay

To perform migration assays, T24 or 5637 cells were cultured with different concentrations of SC66 (0, 5 and 10 µmol/L) for 24 h, and cells were then trypsinized and resuspended in serum‐free medium. After adjusting the cell density, 200 μl of serum‐free medium containing 8 × 10^3^ cells was added to the upper chamber (8‐mm pore size, Corning), and 600 μl of RPMI‐1640 medium containing 10% FBS was added to the lower chamber. After culturing in an incubator (37°C and 5% CO_2_) for 24 h, non‐migrated cells in the upper chamber were removed with a cotton swab, and migrated cells were fixed with 4% paraformaldehyde. Finally, cells were stained with 0.5% crystal violet solution for 15 min, washed and air‐dried, and an inverted microscope (Olympus) was then used to acquire images of the migrated cells. Cells were counted in five random fields, and the average value was calculated. To perform the invasion assay, Matrigel and serum‐free medium were first mixed at a ratio of 1:8. Then, 80 µl of the mixed solution was added to the upper chamber, and the remaining experimental steps were the same as the migration experiment.

### 2.8 Cell cycle assay

After treating T24 and 5637 cells with different concentrations of SC66 for 24 h, cells were collected by trypsinization and washed three times with PBS. According to the instructions of the kit, cells were fixed with 75% of cold ethanol at −20°C overnight and washed again with PBS. Subsequently, cells were incubated with RNase‐containing PBS for 30 min and stained with propidium iodide (PI) for 15 min. Finally, a CytoFLEX flow cytometer (Beckman Coulter Life Sciences) was used to evaluate the cell cycle, and FlowJo version 10 software was used to analyse and process the results. Each experiment was repeated three times.

### 2.9 Apoptosis assay

An Annexin V‐PE/7‐ADD kit (Becton Dickinson) was used to detect the level of apoptosis. In brief, after treating T24 and 5637 cells with different concentrations of SC66, cells were trypsinized, collected and washed three times with cold PBS. Cells were then resuspended in 500 µl of 1× binding buffer, and 5 µl of Annexin and 5 µl of PI were added to the binding buffer and incubated in the dark for 15 min. Finally, flow cytometry (BD FACSCalibur) was used to detect the apoptotic rate of the cells. Each experiment was repeated three times.

### 2.10 TUNEL detection

The TUNEL assay was performed using the In Situ Apoptosis Detection kit (Roche Applied Science) according to the manufacturer's protocol. In brief, cell and tissue samples were prepared, and apoptotic cells were accurately labelled by terminal transferase‐medicated dUTP nick‐end labelling. Finally, an upright fluorescence microscope (Olympus BX51) was used to acquire images. In tissue samples, apoptotic cell nuclei were stained brown, and negative cell nuclei were stained blue.

### 2.11 Western Blot analysis

Cell and tissue samples were collected and lysed on ice in RIPA buffer (Beyotime) containing 0.1 mM PMSF and a protease inhibitor (Roche) for approximately 30 min. After centrifugation of the lysate, the supernatant was collected, and the protein concentration was determined by the BCA method (Beyotime). The samples were subjected to 12% or 15% SDS‐PAGE, and the protein was transferred to a PVDF membrane, which was then blocked with 5% skimmed milk at room temperature for 1 h. After washing three times with TBS‐T, the PVDF membrane was incubated with the corresponding antibody overnight at 4°C. After washing three times with TBS‐T, the membrane was then incubated with goat anti‐mouse IgG (SA00001‐1, Proteintech) or goat anti‐rabbit IgG (SA00001‐2, Proteintech) at room temperature for 1 h. Finally, a ChemiDoc™ Touch Imaging System (BIO‐RAD) was used to scan protein bands and acquire images, and ImageJ software was used to analyse the results. The experiment was repeated three times for each group.

### 2.12 Immunofluorescence staining

T24 and 5637 cells were seeded in a six‐well plate with coverslips at a density of 5 × 10^4^ per well and then treated with 10 µmol/L SC66 for 12 h. The control group was left untreated. Cells were fixed in 4% paraformaldehyde for 15 min, permeabilized with 0.5% Triton‐X‐100 for 5 min, blocked with 1% bovine serum albumin V (Service Bio) for 1 h and incubated with diluted primary antibodies (SNAI1, vimentin, Bcl‐2 and β‐catenin) at 4°C overnight. After sufficient washing, cells were incubated with the corresponding secondary antibody (Proteintech) were incubated for 1 h in the dark at room temperature. Finally, cells were counterstained with DAPI (ab104139, Abcam), and the coverslip was fixed on a glass slide. An upright fluorescence microscope (Olympus BX51) was used to obtain the images.

### 2.13 Immunohistochemistry staining

First, tumour tissue was fixed with formalin, embedded in paraffin and cut into tissue sections with a thickness of 4 µm. The tissue sections were then deparaffinized and rehydrated. After treatment with 0.3% hydrogen peroxide in methanol, the sections were blocked in 1% BSA for 30 min. The sections were then incubated with the corresponding primary antibodies (P‐AKT, P‐GSK‐3β, vimentin, Bax and β‐catenin) at 4°C overnight. After washing thoroughly with PBS, the sections were incubated with horseradish peroxidase (HRP)‐conjugated IgG for 30 min and then incubated with DAB (Vector Laboratories). Finally, the sections were counterstained with haematoxylin, and images were acquired using a microscope (Olympus BX51).

### 2.14 Luciferase assays

T24 and 5637 cells were seeded on a six‐well plate at a density of 5 × 10^4^ cells per well. After treating the cells with different concentrations of SC66 for 12 h, cells were transfected with the TCF/LEF‐1 luciferase reporter plasmid (Qiagen GmbH) according to the manufacturer's instructions. Twenty‐four hours after transfection, the Dual‐Luciferase^®^ Reporter Assay System (Promega, Madison) was used to measure the activity of Fly and Renilla luciferase. Each test was repeated three times.

### 2.15 Animal experiments

**FIGURE 1:**
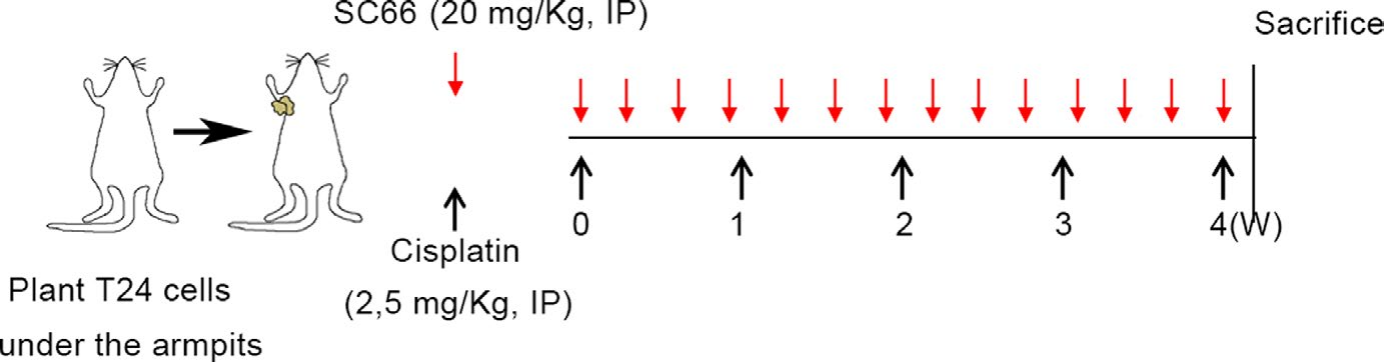
Schematic diagram of nude mouse modelling and drug treatment

Five‐week‐old Balb/c nude mice were purchased from the Centre of Experimental Animals at Wuhan University Medicine College (Hubei, China), and this study was approved by the Animal Experiment Ethics Committee of Wuhan University. All nude mice were kept in a standard temperature‐controlled isolation package and allowed to drink and eat freely. T24 cells (2 × 10^6^) resuspended in 100 μl of PBS were inoculated into the right axilla of each nude mouse. When the tumour volume reached about 100 mm^3^, the tumour‐bearing mice were randomly divided into four groups (*n* = 6) as follows: control group (DMSO, i.p. injected every 2 days), cisplatin group (2.5 mg/kg cisplatin, i.p. injected once a week), SC66 group (20 mg/kg SC66, i.p. injected every 2 days) and the SC66 + cisplatin group (2.5 mg/kg cisplatin was injected intraperitoneally once a week, and 20 mg/kg SC66 was injected intraperitoneally every 2 days). The drug treatment lasted for 4 weeks, during which the tumour volume was measured every 3 days (*V* = *L* × *W* 2 × 1/2). Nude mice were sacrificed 4 days after the last drug treatment, and tumour tissues were collected for follow‐up experiments (Figure 1).

### 2.16 Statistical analysis

All statistical graphs in this manuscript were constructed using GraphPad Prism 5.0 software. SPSS 19.0 software (SPSS Inc.) was used to perform the one‐way analysis of variance (ANOVA) to evaluate whether the differences between the experimental data of each group were statistically significant. *p* < 0.05 was considered significant. All data are expressed as the mean ± SD based on three independent experiments.

## 3 RESULTS

### 3.1 SC66 inhibits the proliferation of BC cells in vitro

**FIGURE 2:**
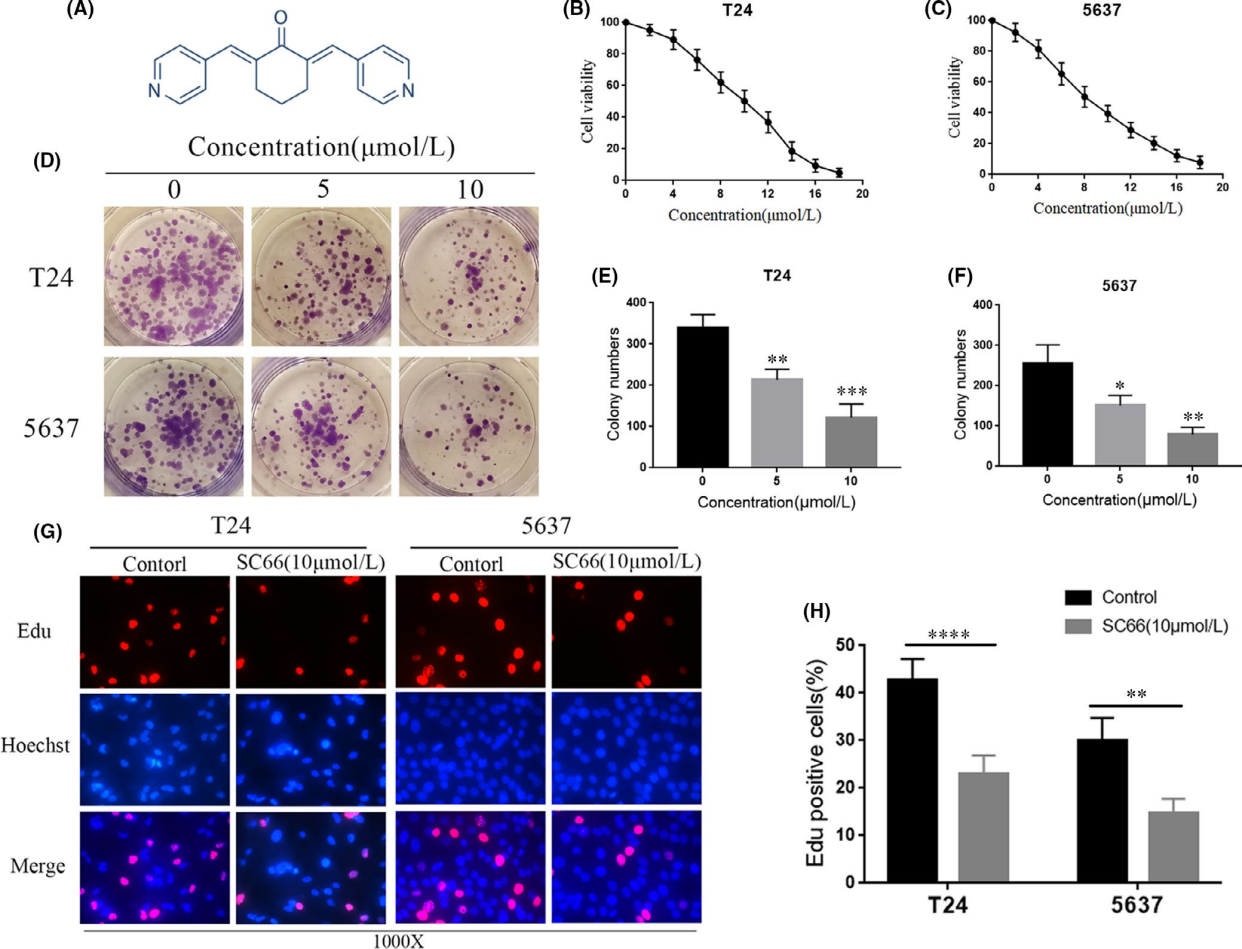
SC66 inhibits the proliferation of T24 and 5637 cells in vitro. (A) A schematic diagram showing the chemical structure of SC66. (B, C) The viability of T24 and 5637 cells was detected using a CCK‐8 assay after treatment with different concentrations of SC66. (D–F) SC66 significantly inhibited the colony formation of T24 and 5637 cells. (G) The EdU assay showed that the DNA synthesis of cells treated with SC66 was significantly reduced compared to that of the control group. (H) Quantitative analysis of the percentage of EdU‐positive cells. All data are expressed as the mean ± SD based on three independent experiments. *p < 0.05, **p < 0.01, ***p < 0.001, ****p < 0.0001

SC66 is a new type of allosteric AKT inhibitor, and the chemical formula of SC66 is shown in Figure 2A. To explore the effect of SC66 on the proliferation activity of BC cells, the cell viability of T24 and 5637 cells was tested at different drug concentrations using the CCK‐8 assay. SC66 significantly inhibited the proliferation of T24 and 5637 cells, and the degree of inhibition was positively correlated with the drug concentration. The IC50 values of SC66 in T24 and 5637 cells were approximately 10 and 8 µmol/L, respectively (Figure 2B,C). In addition, the results of the colony formation assay showed that as the concentration of SC66 increased, the number and size of colonies significantly decreased (Figure 2D–F). In addition, an EdU‐DNA synthesis assay was also used to detect cell proliferation activity, and the results showed that the percentage of EdU‐positive cells in the control group was significantly higher than that in the SC66 group (Figure 2G,H). In summary, these results indicated that the proliferation of T24 and 5637 cells is significantly inhibited after SC66 treatment and that the degree of inhibition is positively correlated with the drug concentration of SC66.

### 3.2 SC66 inhibits the EMT of BC cells in vitro

**FIGURE 3:**
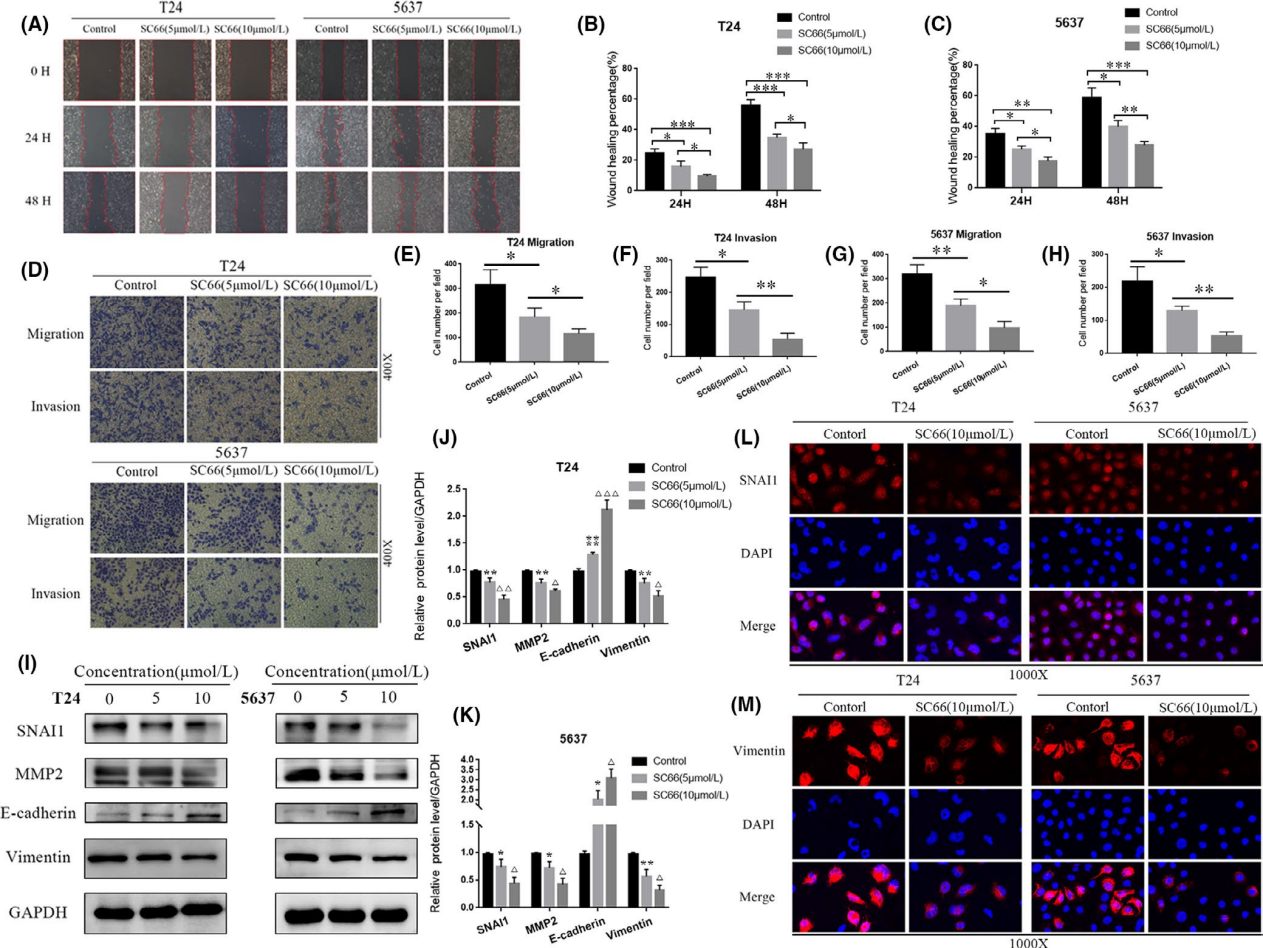
SC66 inhibits the EMT in a concentration‐dependent manner in T24 and 5637 cells in vitro. (A–C) The migration of T24 and 5637 cells was inhibited by SC66 as measured by a wound‐healing assay. (D–H) The migration and invasion of T24 and 5637 cells were inhibited by SC66 as measured by a Transwell assay. (I–K) Expression levels of EMT‐related proteins, including SNAI1, MMP2, vimentin and E‐cadherin, in normal BC cells and BC cells treated with SC66. (L, M) Representative fluorescence images of SNAI1 and vimentin in T24 and 5637 cells. All data are expressed as the mean ± SD based on three independent experiments. *p < 0.05, **p < 0.01, ***p < 0.001 and ****p < 0.0001 versus the control group; ^Δ^ *p* < 0.05, ^ΔΔ^ *p* < 0.01 and ^ΔΔΔ^ *p* < 0.001 versus the SC66 (5 μmol/L) group

Studies have shown that epithelial‐mesenchymal transition (EMT) plays an important role in promoting tumour invasion and metastasis in many cancers. Based on the inhibitory effect of SC66 on the proliferation of BC cells, we investigated whether SC66 also inhibits the EMT of BC cells. A wound‐healing assay was performed to quantify the effect of SC66 on the motility of BC cells, and the results showed that SC66 significantly inhibited wound healing (within the red line area) in a concentration‐dependent manner (Figure 3A–C). The percentage of wound healing in the control group was significantly higher than that of BC cells treated with SC66. In addition, a Transwell assay was used to evaluate cell invasion and migration. The results showed that SC66 significantly inhibited the migration ability of T24 and 5637 cells. Consistent with this result, the invasive ability of BC cells treated with SC66 was significantly lower than that of control cells (Figure 3D–H). Western blot analysis verified the effect of SC66 on the EMT of T24 and 5637 cells at the protein level. The results showed that SC66 treatment significantly inhibited the expression levels of EMT‐related proteins, including SNAI1, MMP2 and vimentin, but upregulated the expression levels of E‐cadherin (Figure 3I–K). Moreover, immunofluorescence analysis showed that the expression levels of SNAI1 and vimentin in T24 and 5637 cells treated with SC66 were significantly downregulated (Figure 3L,M). In summary, these results indicated that the EMT of BC cells is reversed after SC66 treatment.

### 3.3 SC66 arrests the BC cell cycle in the G0/G1 phase in vitro

**FIGURE 4:**
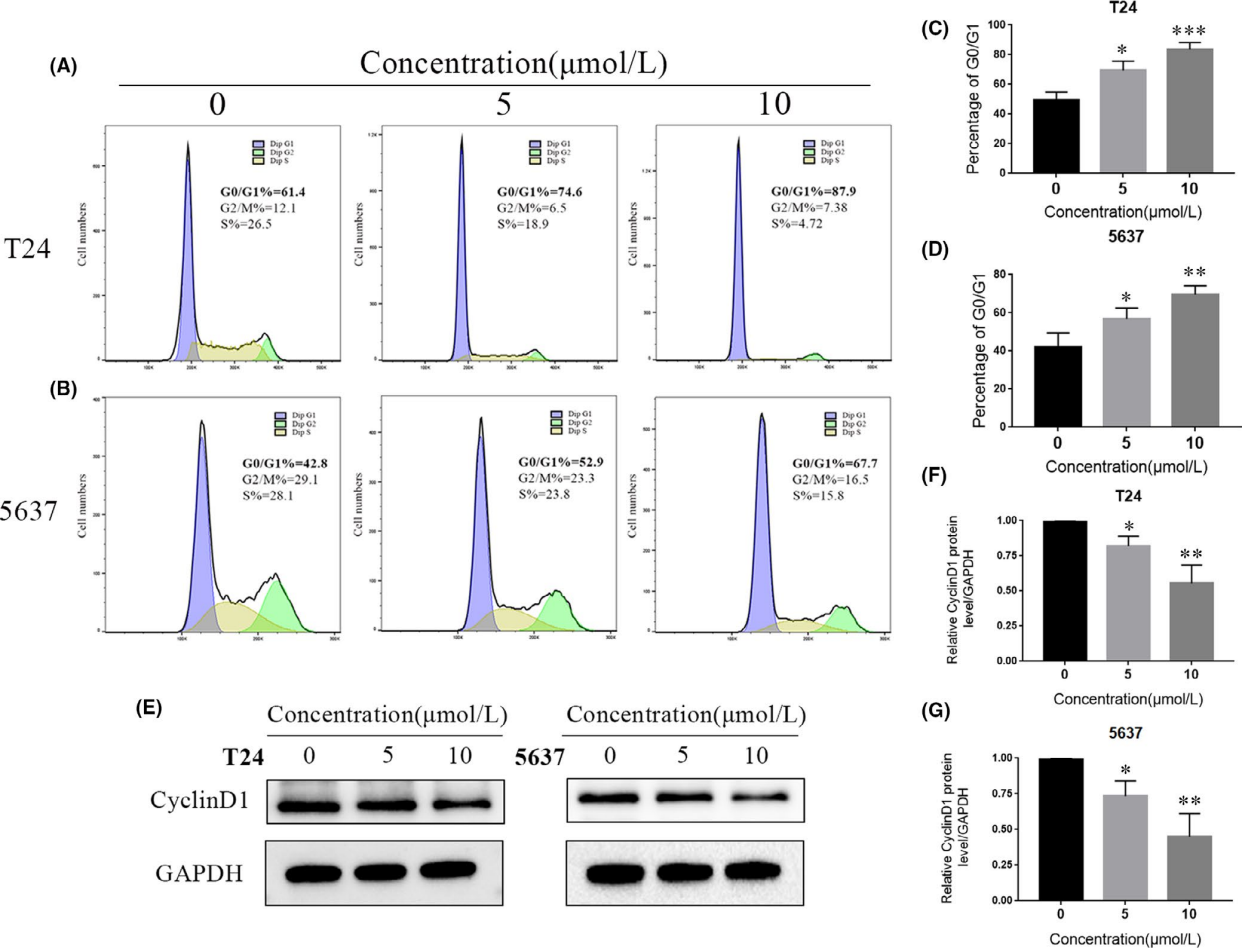
SC66 arrests the cell cycle at G0/G1 phase in T24 and 5637 cells. (A–D) After treatment with different concentrations of SC66, the cycle distribution in T24 and 5637 cells was measured by flow cytometry analysis. (E–G) Representative protein bands and quantitative analysis results of cyclin D1 in T24 and 5637 cells. All data are expressed as the mean ± SD based on three independent experiments. *p < 0.05, **p < 0.01 and ***p < 0.001 versus the 0 μmol/L group

Dysregulation of the cell cycle leads to uncontrolled proliferation of tumour cells. To explore whether SC66 affects the cell cycle to inhibit tumour growth, flow cytometry analysis was used to detect the cycle distribution of T24 and 5637 cells after SC66 treatment. The results showed that after SC66 treatment, the percentage of cells in the G0/G1 phase significantly increased (Figure 4A–D). Consistent with this result, the expression level of cyclin D1, a protein that drives the cell cycle from G1 to S phase, was significantly downregulated after SC66 treatment (Figure 4E–G, Data S1).

### 3.4 SC66 induces BC cells apoptosis in vitro

**FIGURE 5:**
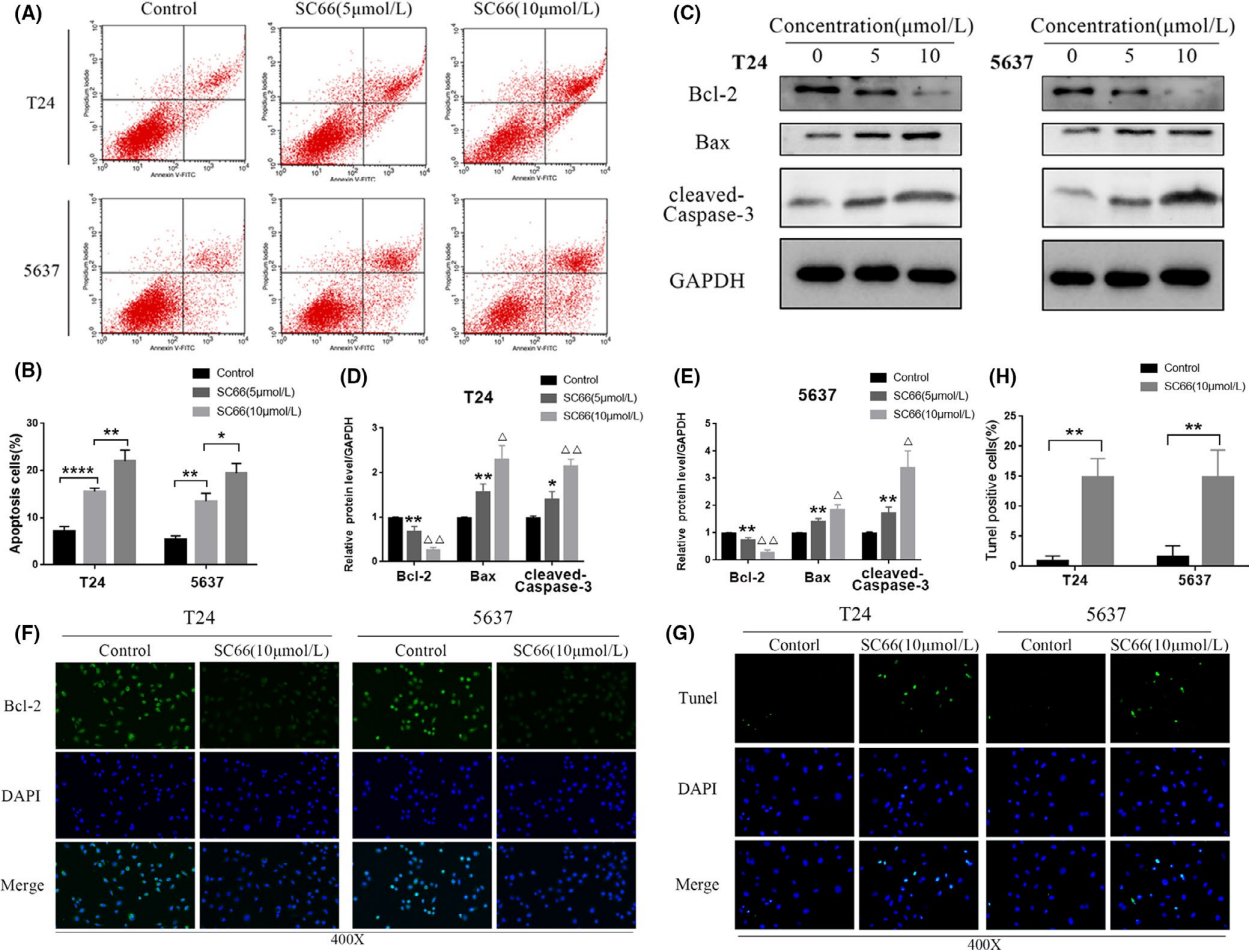
SC66 induces apoptosis of T24 and 5637 cells. (A, B) After treating BC cells with different concentrations of SC66 for 24 h, flow cytometry was used to detect the level of apoptosis in each group. (C–E) Representative bands of apoptosis‐related proteins, including Bcl‐2, Bax and cleaved‐Caspase‐3, and the quantitative results of their expression levels. (F) Representative fluorescence images of Bcl‐2 in T24 and 5637 cells. (G, H) Representative image of TUNEL staining and quantitative analysis of the proportion of TUNEL‐positive cells in each group. All data are expressed as the mean ± SD based on three independent experiments. *p < 0.05, **p < 0.01 and ****p < 0.0001 versus the control group; ^Δ^ *p* < 0.05 and ^ΔΔ^ *p* < 0.01 versus the SC66 (5 μmol/L) group

In addition, we further explored whether SC66 achieves its antitumour effect by inducing apoptosis. After different concentrations of SC66 were used to treat BC cells for 24 h, flow cytometry analysis was used to detect the level of apoptosis in each group. The results showed that SC66 induced apoptosis of T24 and 5637 cells in a concentration‐dependent manner (Figure 5A,B). Furthermore, Western blot analysis showed that the expression levels of proapoptotic proteins, such as Bax and cleaved‐Caspase‐3, were significantly upregulated after SC66 treatment (Figure 5C–E). However, the expression level of the antiapoptotic protein, Bcl‐2, was significantly downregulated according to Western blot analysis and immunofluorescence staining analysis (Figure 5C–F). Consistent with these results, TUNEL staining assays showed that the percentage of TUNEL‐positive cells in BC cells treated with SC66 was higher than that of the control group (Figure 5G,H). In summary, the above results indicated that SC66 induces apoptosis in T24 and 5637 cells.

### 3.5 SC66 inhibits AKT/β‐catenin signalling pathways in BC cells in vitro

**FIGURE 6:**
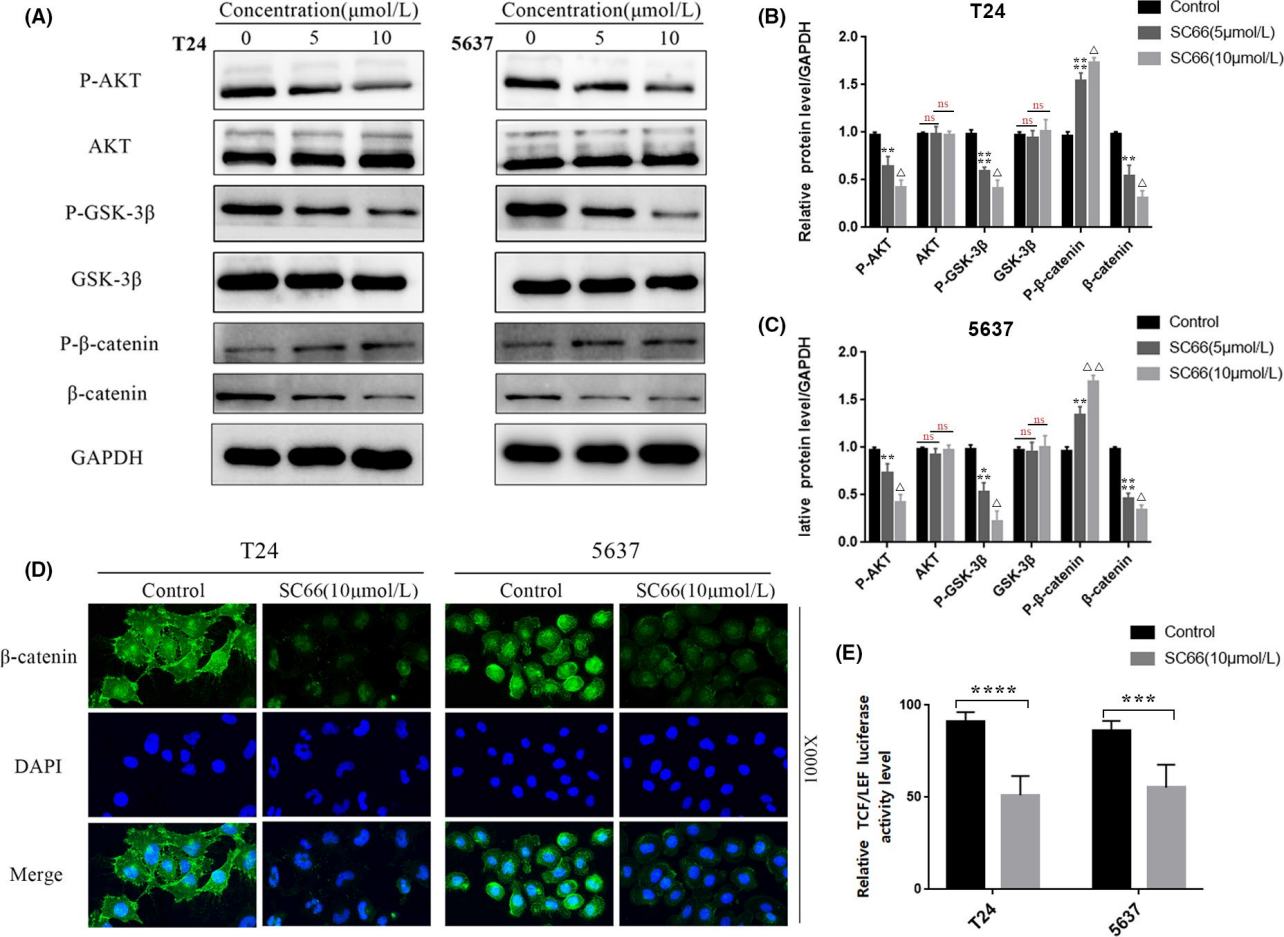
SC66 exerts antitumour effects through AKT/β‐catenin signalling pathways in T24 and 5637 cells. (A–C) Representative bands of AKT/β‐catenin signalling pathway‐related proteins, including AKT, GSK‐3β, β‐catenin and their respective phosphorylated forms, and quantitative analysis of their expression levels after SC66 treatment. (D) After SC66 treatment, the expression level of β‐catenin in T24 and 5637 cells was measured by immunofluorescence. (E) After 24 h of SC66 treatment, the relative TCF/LEF luciferase activity in T24 and 5637 cells was detected and statistically analysed. All data are expressed as the mean ± SD based on three independent experiments. n.s., no significance; **p < 0.01, ***p < 0.001 and ****p < 0.0001 versus the control group; ^Δ^ *p* < 0.05 and ^ΔΔ^ *p* < 0.01 versus the SC66 (5 μmol/L) group

To explore whether the antitumour effect of SC66 is achieved by inhibiting the AKT signalling pathway, we used Western blotting to detect the expression levels of proteins, including AKT, GSK‐3β, β‐catenin and their respective phosphorylated forms, in BC cells treated with different concentrations of SC66. As the concentration of SC66 increased, the expression levels of P‐AKT and P‐GSK‐3β gradually decreased, while the expression levels of total AKT and GSK‐3β did not significantly change. In contrast, the expression of P‐β‐catenin in BC cells treated with SC66 significantly increased, while the expression of β‐catenin gradually decreased (Figure 6A–C). In addition, we used immunofluorescence to detect the expression level of β‐catenin, and the results were consistent with the results of Western blot analysis (Figure 6D). Furthermore, we used TCF/LEF luciferase reporter analysis to explore the effect of SC66 on β‐catenin‐mediated TCF/LEF activity. The results showed that the luciferase activity in T24 and 5637 cells decreased significantly after SC66 treatment, indicating SC66 significantly inhibits the transcriptional activity of TCF/LEF (Figure 6E). The above results indicated that SC66 exerts its antitumour effect by inhibiting AKT/β‐catenin signalling pathways in T24 and 5637 cells.

### 3.6 Enhancing β‐catenin activity rescues the anti‐BC effect of SC66

**FIGURE 7:**
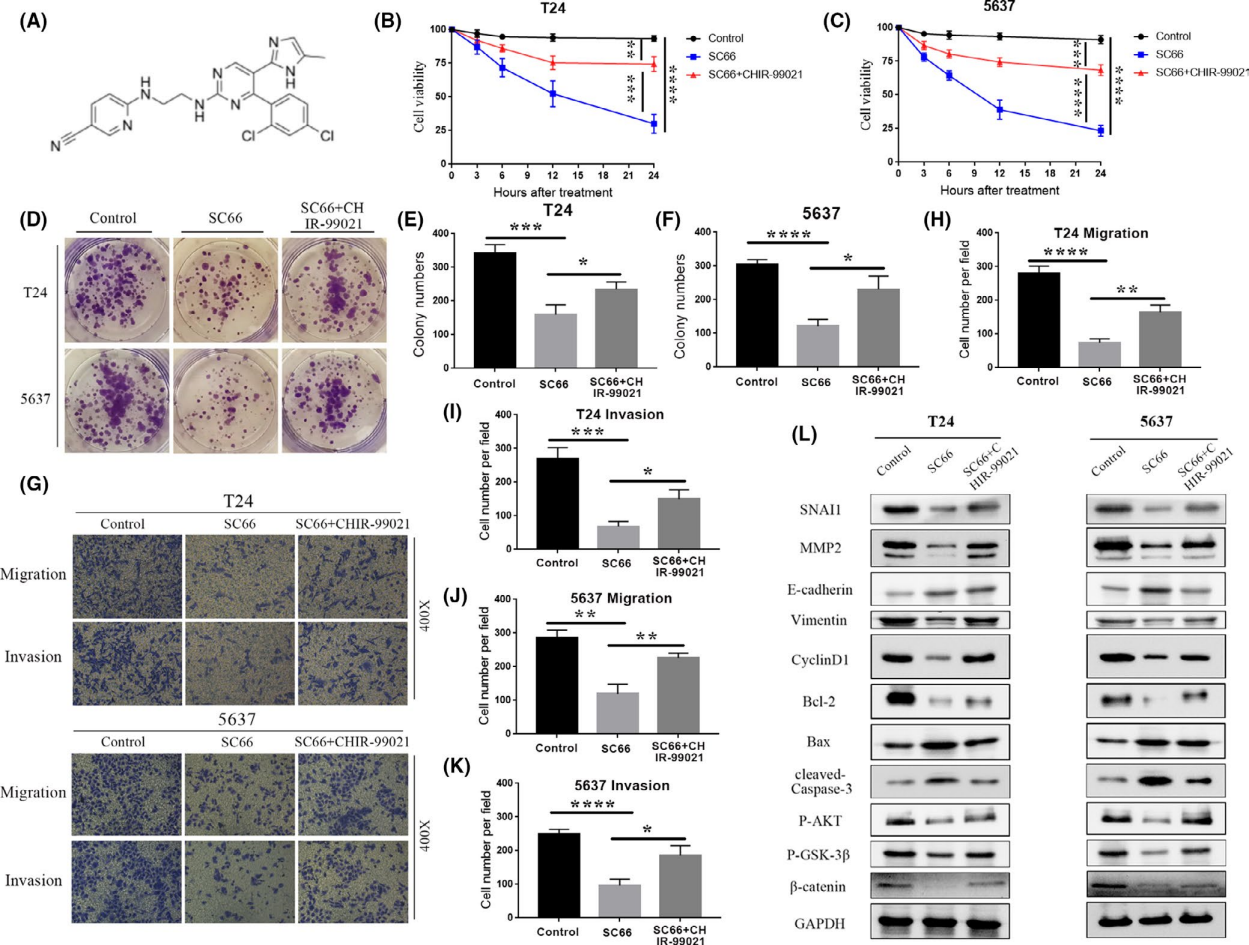
CHIR‐99021 reduces the antitumour effect of SC66 by activating Wnt/β‐catenin signalling pathways in T24 and 5637 cells. (A) A schematic diagram showing the chemical structure of CHIR‐99021. (B, C) The viability of T24 and 5637 cells was detected using a CCK‐8 assay after treatment with SC66 and CHIR‐99021. (D–F) CHIR‐99021 reduced the inhibitory effect of SC66 on colony formation of T24 and 5637 cells. (G–K) CHIR‐99021 rescued the inhibitory effect of SC66 on T24 and 5637 cell migration and invasion. (L) Representative bands of major proteins after treatment with SC66 and CHIR‐99021. All data are expressed as the mean ± SD based on three independent experiments. *p < 0.05, **p < 0.01, ***p < 0.001 and ****p < 0.0001

**FIGURE 8:**
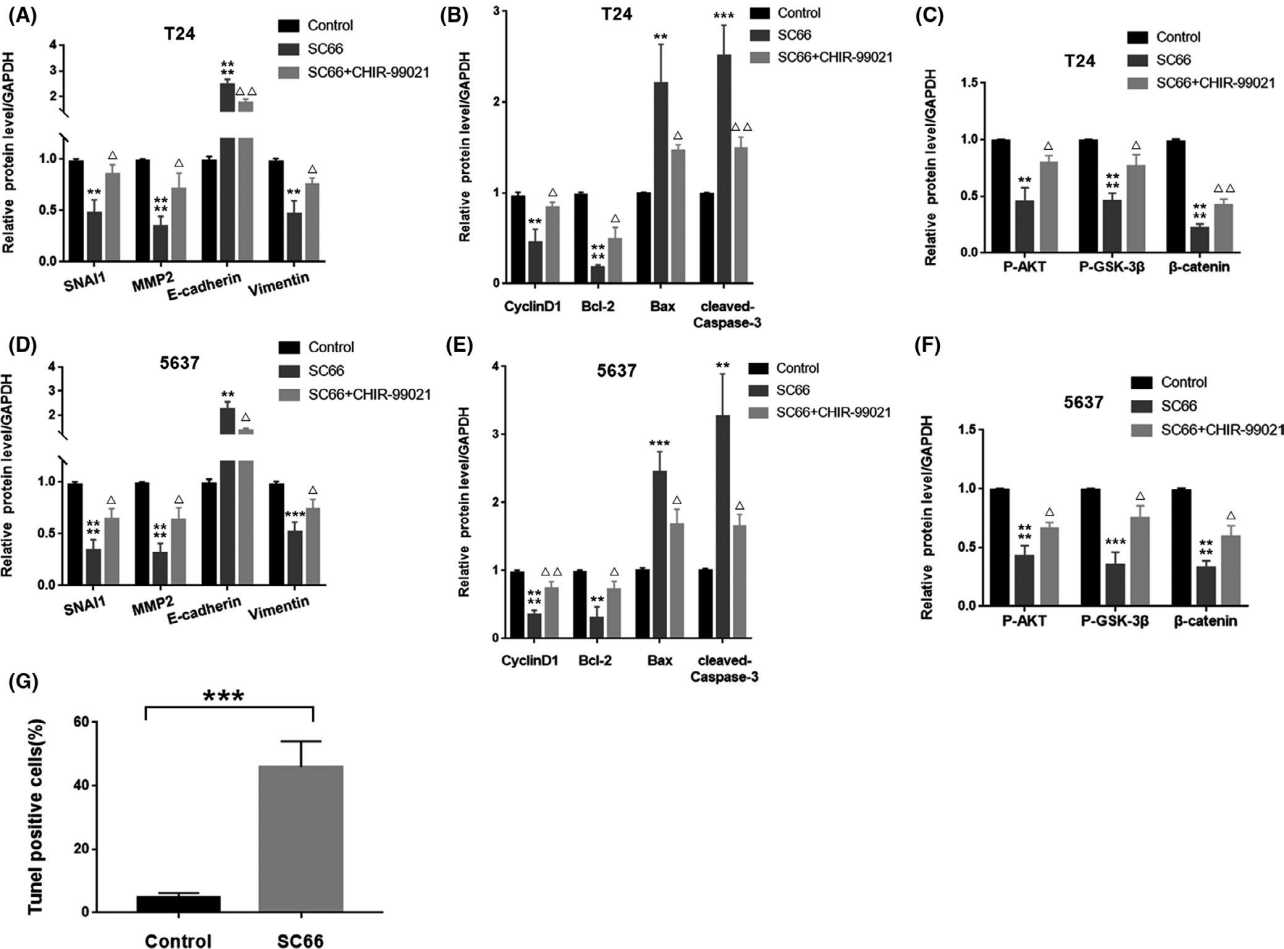
CHIR‐99021 reduces the antitumour effect of SC66 in T24 and 5637 cells. (A‐F) Quantitative analysis results of key protein expression levels (G) After intraperitoneal injection of SC66, the percentage of TUNEL‐positive cells. All data are expressed as the mean ± SD based on three independent experiments. **p < 0.01, ***p < 0.001 and ****p < 0.0001 versus the control group; Δ*p* < 0.05 and ΔΔ*p* < 0.01 versus the SC66 group

To further prove that SC66 exerts antitumour effects through AKT/β‐catenin signalling pathways, we selected CHIR‐99021 for rescue experiments. CHIR‐99021 is a selective GSK‐3β inhibitor and a potent Wnt/β‐catenin signalling pathway activator. The chemical formula of CHIR‐99021 is shown in Figure 7A. After SC66 treatment of BC cells for 24 h, CHIR‐99021 was added, and the follow‐up experiment was performed. The CCK‐8 assays showed that the cell viability of the SC66 + CHIR‐99021 group was higher than that of the SC66 group (Figure 7B,C). Colony formation assays also confirmed the above results, indicating that CHIR‐99021 alleviates the inhibitory effect of SC66 on T24 and 5637 cell proliferation (Figure 7D–F). In addition, Transwell assays also confirmed that CHIR‐99021 rescued the inhibitory effect of SC66 on BC cell migration and invasion (Figure 7G–K). After CHIR‐99021 treatment, Western blot analysis confirmed that the expression levels of P‐AKT, P‐GSK‐3β and β‐catenin in the SC66 + CHIR‐99021 group were significantly upregulated compared to those in the SC66 group (Figures 7L and 8A–F). In addition, the EMT‐inhibiting and proapoptotic effects of SC66 were also attenuated after CHIR‐99021 treatment (Figures 7L and 8A–F). Together, these results confirmed that CHIR‐99021 reduces the antitumour effect of SC66 by activating Wnt/β‐catenin signalling pathways.

### 3.7 SC66 combined with cisplatin further inhibits the growth of human BC in a tumour‐bearing nude mouse model

**FIGURE 9:**
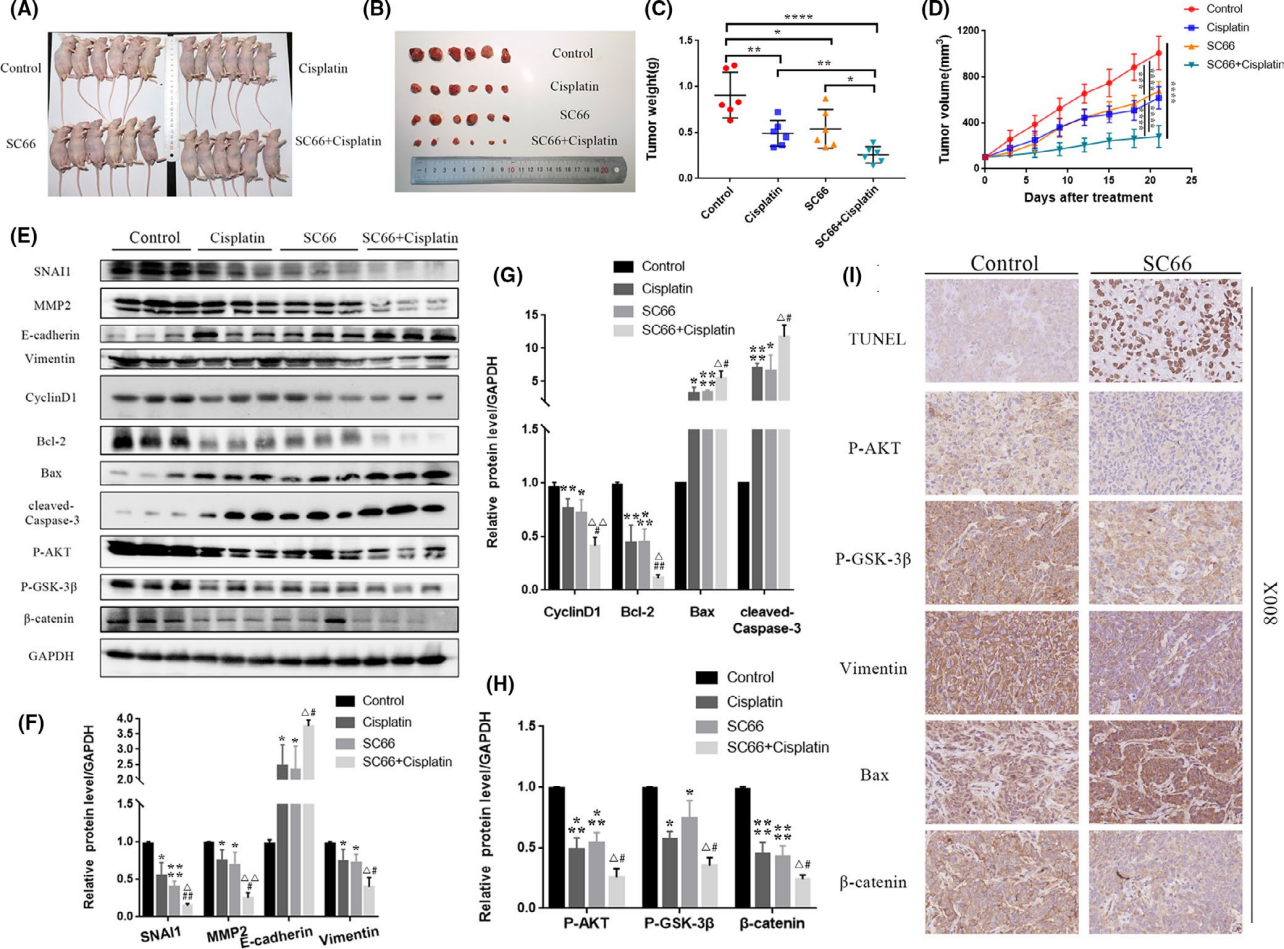
SC66 and cisplatin suppress tumour growth in nude mouse models. (A) Images of nude mice with tumours undergoing different treatments. (B) Representative images of dissected tumours treated with SC66, cisplatin and SC66 + cisplatin. (C–D) Tumour weight and volume after treatment with SC66 or cisplatin alone or combined therapy. (E–H) Representative bands of major proteins in different treatment groups and quantitative analysis of protein expression levels. (I) TUNEL staining images of the control group and SC66 group as well as representative images of IHC staining of P‐AKT, P‐GSK‐3β, vimentin, Bax and β‐catenin. All data are expressed as the mean ± SD based on three independent experiments. *p < 0.05, **p < 0.01, ***p < 0.001 and ****p < 0.0001 versus the control group; ^Δ^ *p* < 0.05 and ^ΔΔ^ *p* < 0.01 versus the cisplatin group; ^#^ *p* < 0.05 and ^##^ *p* < 0.01 versus the SC66 group

To evaluate the antitumour ability of SC66 in vivo, we established a T24 xenograft tumour model in nude mice. Because cisplatin is an important chemotherapy drug for the treatment of advanced BC, we explored the efficacy of SC66 combined with cisplatin. Both SC66 and cisplatin significantly inhibited tumour growth. Specifically, the weight and volume of tumours in the SC66 group and the cisplatin group were significantly reduced, and the combined treatment group showed the best curative effect (Figure 9A–D). In addition, we used Western blot analysis, TUNEL staining assays and immunohistochemical staining to further confirm that SC66 exerted its antitumour effect by inhibiting EMT, affecting cell cycle distribution and promoting cell apoptosis by inhibiting AKT/β‐catenin signalling pathways in vivo (Figures 8G and 9E–I). Similar to the previous results, the combined treatment group achieved the best antitumour effect.

## 4 CONCLUSION

The data from GLOBOCAN show that an estimated 570,000 people were diagnosed with BC in 2020, accounting for nearly 3% of all newly diagnosed cancers.^1^ BC originates from the urothelium and is divided into non‐muscle‐invasive bladder cancer and muscle‐invasive bladder cancer. Surgical treatment is currently the main treatment for local BC.^25^, ^26^ Recent studies have shown that compared with cystectomy alone, platinum‐based neoadjuvant chemotherapy combined with cystectomy has a 33% reduction in the risk of death,^27^ and the 10‐year survival rate is increased by 6%.^28^ However, the drug resistance and adverse reactions of chemotherapeutics often limit the chemotherapy for BC.^29^, ^30^

Immortal cell proliferation is the main feature of tumour cells and is related to cell cycle disorders.^31^, ^32^ Our study confirmed that SC66 inhibited the proliferation of T24 and 5637 cells in a concentration‐dependent manner. Flow cytometry analysis revealed that SC66 inhibited BC cell proliferation by blocking the cell cycle in the G0/G1 phase. Cyclin D1 is an important regulatory protein of the cell cycle that drives cells to transition from G0/G1 to S phase by binding to CDK4 or CDK6 to form a cyclin D1‐CDK4/6 complex.^33^, ^34^, ^35^ In this study, SC66 treatment significantly inhibited the expression of cyclin D1 in BC cells in vitro. Animal studies further confirmed this result. The weight and volume of tumour tissue in SC66‐treated nude mice were significantly reduced, and the expression level of cyclin D1 was significantly downregulated. These results indicated that SC66 inhibits the proliferation of BC cells by blocking the cell cycle in the G0/G1 phase and that this inhibition is related to the inhibition of cyclin D1 expression.

EMT is the process by which tumour epithelial cells acquire the phenotypic characteristics of mesenchymal cells and has been confirmed to be involved in tumour cell migration and invasion.^36^, ^37^, ^38^ We observed a decline in the migration and invasion ability of BC cells treated with SC66 by Transwell and wound‐healing assays in vitro. Western blot analysis confirmed these results. The expression of EMT marker proteins, including SNAI1, MMP2 and vimentin, decreased in the SC66 group, and the expression of E‐cadherin was increased in the SC66 group in vivo and in vitro. These results showed that SC66 inhibits the migration and invasion of BC cells by inhibiting EMT.

Apoptosis is programmed cell death controlled by genes, and it is one of the important mechanisms for maintaining cell proliferation and death. Studies have shown that apoptosis is an important means by which the human body fights tumour proliferation.^39^, ^40^, ^41^ The mechanism of apoptosis is complicated, and mitochondrial‐induced apoptosis is one of the main mechanisms.^42^ In this process, Bax is activated and upregulates mitochondrial membrane permeability, and cytochrome C is released in large quantities and activates caspase‐9 and caspase‐3, ultimately leading to cell apoptosis. In the present study, flow cytometry analysis confirmed that SC66 promoted BC cell apoptosis in a concentration‐dependent manner. Western blot analysis confirmed that the expression level of cleaved‐Caspase‐3 and the Bax/Bcl‐2 ratio was upregulated in vivo and in vitro. In addition, TUNEL analysis yielded the same result. These results indicated that SC66 induces BC cell apoptosis by activating the mitochondrial apoptosis pathway.

**FIGURE 10:**
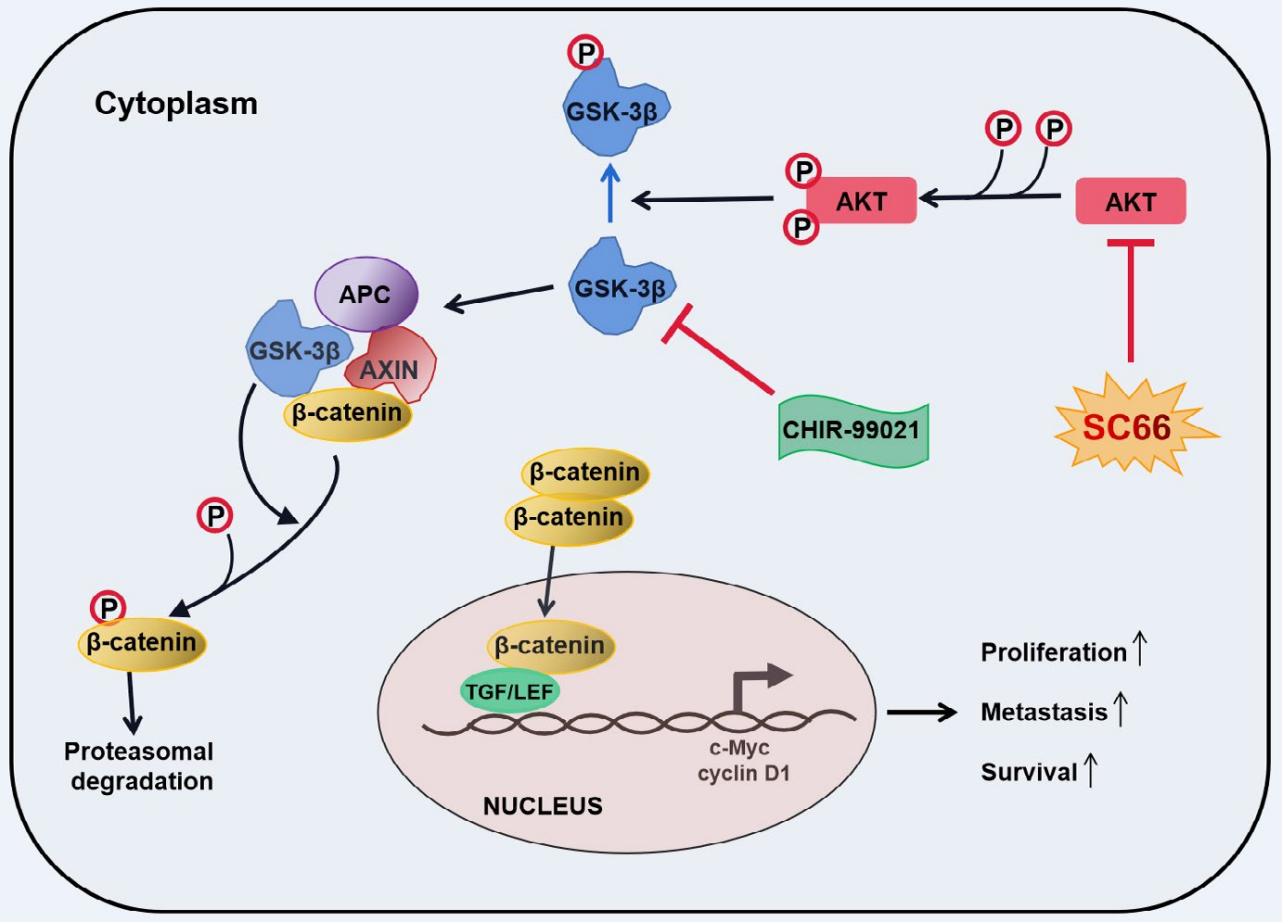
The schematic diagram explains the molecular mechanism of SC66's anti‐cancer effect. In short, SC66 regulates the expression of downstream target genes through the AKT/GSK‐3β/β‐catenin signalling pathway to inhibit the proliferation, migration, invasion of BC cells and induce apoptosis

After confirming that SC66 inhibits EMT‐mediated metastasis, promotes apoptosis and affects the cell cycle, we further explored the molecular mechanism of the antitumour effect of SC66. β‐catenin is a multifunctional protein that plays an important role in maintaining normal physiological functions.^43^ Under normal circumstances, the destruction complex composed of APC, axin (axin‐1 and axin‐2), GSK‐3β and casein kinase 1α (CK1α) recruits β‐catenin in the cytoplasm and mediates the phosphorylation, final ubiquitination and proteasomal degradation of β‐catenin to isolate β‐catenin from the nucleus. In tumour cells, however, the Wnt/β‐catenin signal transduction pathway is activated,^44^, ^45^ and the destruction complex is also destroyed, resulting in loss of the GSK‐3β‐dependent phosphorylation of β‐catenin. Subsequently, β‐catenin enters the nucleus in an active form and interacts with transcription factors of the TCF/LEF‐1 family, thereby promoting the transcription of downstream target genes, such as c‐Myc and cyclin D1y^46^, ^47^ (Figure 10). In the present study, SC66 treatment promoted the GSK‐3β‐dependent phosphorylation of β‐catenin in vivo and in vitro and the expression of P‐β‐catenin was upregulated. In addition, TCF/LEF luciferase reporter analysis confirmed that luciferase activity in T24 and 5637 cells decreased significantly after SC66 treatment. Finally, after treatment with CHIR‐99021, an inhibitor of GSK‐3β, the anti‐BC effects of SC66, including EMT inhibition, apoptosis promotion and cell cycle effects were all reversed. Together, these results confirmed that SC66 exerts its anti‐BC effect through AKT/β‐catenin signalling pathways.

In animal experiments, we also found that the combined use of SC66 and cisplatin exerted a better antitumour effect than SC66 or cisplatin alone. This phenomenon may be related to SC66 increasing the sensitivity of BC cells to cisplatin, but further experiments are needed to prove this conjecture.

In conclusion, the present study confirmed that SC66 exerts its anti‐BC effect in vivo and in vitro through the AKT/β‐catenin signalling pathway, thereby providing a new potential drug for the treatment of BC.

## CONFLICT OF INTEREST

We declare that we have no commercial or associative interests that conflict with the work submitted.

## AUTHOR CONTRIBUTIONS

**Wu Chen:** Data curation (lead); Formal analysis (lead); Validation (lead); Visualization (lead); Writing‐original draft (lead); Writing‐review & editing (lead). **Sheng Zhao:** Data curation (equal); Visualization (equal); Writing‐original draft (equal). **Weimin Yu:** Formal analysis (equal); Investigation (equal); Methodology (equal); Visualization (equal). **Ting Rao:** Formal analysis (equal); Supervision (equal). **Yuan Ruan:** Data curation (equal). **Shaoming Zhu:** Methodology (equal); Validation (equal). **Yuqi Xia:** Data curation (equal). **Hongfei Song:** Funding acquisition (equal). **Fan Cheng:** Funding acquisition (equal).

## CONTRIBUTION TO THE FIELD STATEMENT

The data from GLOBOCAN show that an estimated 570,000 people were diagnosed with BC in 2020, accounting for 3% of all newly diagnosed cancers. Surgery combined with immunotherapy is currently the main treatment for local BC. Studies have shown that compared with cystectomy alone, platinum‐based neoadjuvant chemotherapy combined with cystectomy has a 33% reduction in the risk of death, and the 10‐year survival rate is increased by 6%. However, the drug resistance and adverse reactions of chemotherapeutics often limit the treatment of BC. In recent years, many studies have confirmed that AKT‐targeted therapy significantly inhibits the progression of many cancers, including BC, and SC66 is a new type of AKT inhibitor. This study confirmed that SC66 exerts its anti‐BC effect in vivo and in vitro through the AKT/β‐catenin signalling pathway, specifically including inhibiting EMT‐mediated metastasis, inducing cell cycle redistribution and promoting tumour cell apoptosis. This study provides a new potential drug for the treatment of BC.

## Supporting information

Data S1

## Data Availability

The data supporting the conclusions of this study can be obtained from the corresponding author.
