# Supplementary material for: SC66 inhibits the proliferation and induces apoptosis of human bladder cancer cells by targeting the AKT/β‐catenin pathway
Source: J Cell Mol Med. 2021 Oct 22;25(22):10684–97. doi: 10.1111/jcmm.17005 (PMC8581318; doi:10.1111/jcmm.17005)

Raw data of WB bands of GAPDH in fig3 E

The WB bands of GAPDH of T24 cells in fig3 E


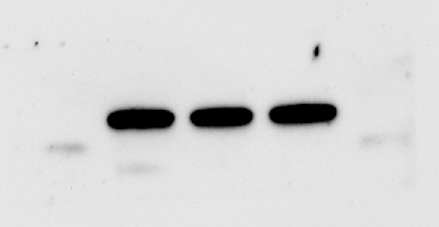

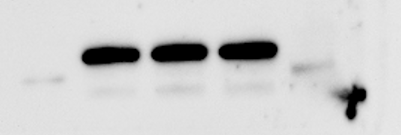

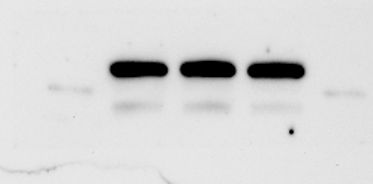


The WB bands of GAPDH of 5637 cells in fig3 E


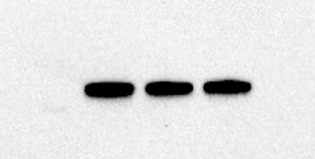

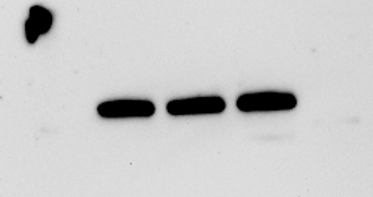


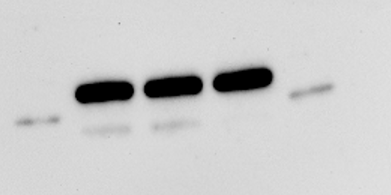

Supplement: Supplementary file 1 — Data S1 [file JCMM-25-10684-s001.docx]
